# Serogrouping and Molecular Characterization of ESBL-Producing Avian Pathogenic *Escherichia coli* from Broilers and Turkeys with Colibacillosis in Algeria

**DOI:** 10.3390/antibiotics14040356

**Published:** 2025-03-31

**Authors:** Nadia Safia Chenouf, Chafik Redha Messaï, Isabel Carvalho, Tamara Álvarez-Gómez, Vanessa Silva, Abdelghani Zitouni, Ahcene Hakem, Patricia Poeta, Carmen Torres

**Affiliations:** 1 Faculty of Natural and Life Sciences, Earth and Universe Sciences, University Mohamed El Bachir El Ibrahimi of Bordj Bou Arreridj, El Anasser, Bordj Bou Arreridj 34000, Algeria; nadia.chenouf@univ-bba.dz (N.S.C.); chafik.messai@univ-bba.dz (C.R.M.); 2 Laboratory for Exploration and Valorization of Steppe Ecosystems (EVES), Department of Biology, Faculty of Natural Sciences and Life, University of Djelfa, Moudjbara Road BP 3117, Djelfa 17000, Algeria; 3 Laboratoire de Biologie des Systèmes Microbiens (LBSM), Ecole Normale Supérieure Cheikh Mohamed El Bachir El Ibrahimi, BP 92, Kouba, Algiers 16000, Algeria; abdelghani.zitouni@g.ens-kouba.dz; 4 Laboratory of Research Health and Animal Production, High National Veterinary School, Issad Abbes Street, Oued Smar, Algiers 16000, Algeria; 5 Microbiology and Antibiotic Resistance Team (MicroART), Department of Veterinary Sciences, University of Trás-os-Montes and Alto Douro (UTAD), 5000-801 Vila Real, Portugal; isabelcarvalho93@hotmail.com (I.C.); ppoeta@utad.pt (P.P.); 6 Area Biochemistry and Molecular Biology, OneHealth-UR Research Group, University of La Rioja, 26006 Logroño, Spain; tamara.alvarez@unirioja.es; 7 LAQV-REQUIMTE, Department of Chemistry, NOVA School of Science and Technology, Universidade Nova de Lisboa, 2829-516 Caparica, Portugal; 8 Agropastoralism Research Center of Djelfa, Djelfa 17000, Algeria; hakem.ahcene@crapast.dz; 9 CECAV—Veterinary and Animal Research Centre, University of Traìs-os-Montes and Alto Douro (UTAD), 5000-801 Vila Real, Portugal; 10 Associate Laboratory for Animal and Veterinary Sciences (AL4AnimalS), 5000-801 Vila Real, Portugal

**Keywords:** Avian colibacillosis, APEC, Serogrouping, *bla*_CTX-M_, ESBL, Algeria

## Abstract

Avian colibacillosis caused by avian pathogenic *Escherichia coli* (APEC) strains is a bacterial disease responsible for enormous economic losses in the poultry industry, due to high mortality rates in farms, antibiotic therapy costs, and seizures at slaughterhouses. The aim of this study was to characterize the serogroups and molecular features of extended spectrum β-lactamase (ESBL)-producing APEC isolates recovered from 248 liver samples of 215 broilers and 33 turkeys with colibacillosis lesions in northeast Algeria. For this, microbiological tests were carried out, according to the recommended standards: *E. coli* isolates were recovered using standard microbiological protocols, and identification was carried out by MALDI-TOF MS. Serogrouping was performed using a rapid agglutination slide and the antisera of three O somatic groups (O1, O2, O78). Antimicrobial susceptibility was determined by the disk diffusion method. PCR assays and sequencing were used to detect antimicrobial resistance genes, integrons, phylogrouping, and MLST. Conjugation experiments were also conducted to determine the transferability of the retrieved ESBL-encoding genes. Overall, 211 (85.1%) APEC isolates were collected (one per positive sample), and 164 (77.7%) of them were typable. The O2 and O1 serogroups were the most detected (46.1% in broiler typable isolates and 61.5% in turkey typable isolates). Seventeen APEC isolates were ESBL-producers and harbored the following genes (number of isolates): *bla*_CTX-M-1_ (14), *bla*_CTX-M-15_ (2), and *bla*_SHV-12_ (1). They belonged to phylogroups D (10 isolates), B1 (6 isolates), and B2 (1 isolate). The MLST of 13 ESBL producers revealed seven STs: ST23, ST38, ST48, ST117, ST131, ST1146, and ST5087. The ESBL-encoding genes were transferred by conjugation among 15 ESBL-producing isolates, and transconjugants acquired either the IncK or IncI1 plasmids. Concerted efforts from all poultry actors are needed to establish surveillance monitoring strategies to mitigate the spread of ESBL-producing isolates implicated in avian colibacillosis.

## 1. Introduction

Avian infections caused by avian pathogenic *Escherichia coli* (APEC), so-called avian colibacillosis (ACL), have been noticed to be among the most reported devastating infectious diseases in poultry husbandry, resulting in weighty economic losses worldwide. These diseases can present as localized or systemic infections, manifesting in various forms across avian species., including colisepticemia, chronic respiratory disease (CRD), yolk sac infection/omphalitis, and osteomyelitis in turkey [1,2].

APEC is known to be either a primary pathogen or occasionally secondary to other.predisposing factors, such as initial respiratory viral (Newcastle disease, infectious bronchitis, avian influenza) and *Mycoplasma* infections [3,4]. APEC strains are classified as a subset of extra-intestinal pathogenic *E*. *coli* (ExPEC)*,* a large group of pathogens with diverse virulent properties. They are serologically categorized into a variety of serogroups according to the serotyping method established by Kauffmann, in which the O antigen structure confers the main basis [5]. Earlier on, most APEC strains associated with ACL were ascribed to three major serogroups, O1, O2, and O78 [6]; nevertheless, although a greater diversity was shown thereafter via WGS [7,8,9,10], serotyping remains an established tool for APEC typing [11,12].

For decades, antibiotics have been extensively used in poultry farming for treatment, prevention, and growth promotion (the latter was banned in European countries in 2006 and later in some other countries). This promoted the dissemination of resistant and/or multidrug-resistant (MDR) bacteria, including APEC strains. Antibiotic resistance (AR) is recognized as a global, escalating health crisis, and poultry is regarded as a worrying source of resistant bacteria that may be transmitted to humans via the food supply chain [13,14]. The production of extended-spectrum β-lactamases (ESBLs) is considered a major player in the AR phenomenon and one of the most significant AR mechanisms used by *E. coli* among other *Enterobacteriaceae* species [15,16]. ESBLs are a group of diverse and rapidly evolving enzymes that inactivate third- and fourth-generation cephalosporins and monobactams but not cephamycin or carbapenems. Multiple ESBL types have been discovered and can be categorized into different families. While TEM- and SHV-type ESBLs are genetically related, CTX-M-type ESBLs have been found to be more diverse and remain the most dominant worldwide [17]. The successful spread of *bla*_CTX-M_-encoding genes is mainly due to their location on conjugative plasmids that may co-harbor other AR genes, resulting in resistance to other multiple antibiotic classes, such as aminoglycosides and fluoroquinolones [15].

In Algeria, poultry constitutes the most dominant livestock, notably in the northeast, and chickens and turkeys were the predominant sources of poultry meat in 2021 (91% and 6.1% of total poultry production, respectively). Although some studies characterized the virulence factors of APEC strains causing ACL in this country [18,19], only a small number investigated the genetic characteristics of antimicrobial-resistant and/or ESBL-producing APEC from diseased animals [20]. Therefore, the objective of this study was to determine the serogrouping and provide an in-depth molecular characterization of ESBL-producing strains collected from broilers and turkeys with colibacillosis lesions in northeast Algeria.

## 2. Results

### 2.1. APEC Isolation, Serogrouping, and Antimicrobial Susceptibility Testing

Out of the 248 liver samples, 211 contained APEC isolates (85.1%) and one isolate per positive sample was selected. These isolates originated from 185 of 215 broilers (86%) and 26 of 33 turkeys (78.8%), as shown in Table 1.

Regarding serogrouping, among the 185 broiler isolates, 143 (77.3%) of them were typable: the O2 serogroup was the predominant one, with 66 isolates (35.7%), followed by O1 with 50 isolates (27%), and then O78 with 27 isolates (14.6%). Moreover, 42 (22.7%) of the broiler APEC isolates were non-typable with these sera reagents. Concerning turkey isolates, 21 (80.8%) were typable: the dominant serogroup was O1 (61.5%), followed by O2 (15.4%), and then O78 (1) (3.8%). Conversely, five turkey isolates (19.2%) were non-typable.

As for antimicrobial susceptibility, high resistance rates were observed in APEC isolates from broilers and turkeys, respectively, towards tetracycline (87.9% and 100%), nalidixic acid (94.6% and 96.1%), ampicillin (81.1% and 100%), trimethoprim/sulfamethoxazole (75.7% and 88.5%), amoxicillin/clavulanic acid (75.7% and 69.2%), and ciprofloxacin (65.4% and 73%). On the contrary, all isolates exhibited susceptibility to imipenem (Table 2). MDR (resistance to at least three antimicrobial classes) was detected in 94% of broiler APEC isolates and in all turkey APEC isolates (Table 3).

### 2.2. Prevalence and Antimicrobial Resistance Genotype of ESBL-Producing APEC Isolates

The ESBL phenotype was detected in 17 out of 211 total APEC isolates (rates of 7% and 15.4% in broiler and turkey samples analyzed, respectively). All ESBL-producing isolates were MDR but showed susceptibility to colistin (MIC ≤ 1 μg/mL). PCR and sequencing assays revealed three ESBL-encoding genes (number of isolates): *bla*_CTX-M-1_ (14), *bla*_CTX-M-15_ (2), and *bla*_SHV-12_ (1) (Table 4). The *bla*_TEM-1_ gene, encoding a widespread β-lactamase, was also found in five ESBL-producing isolates. Regarding resistance to non-β-lactam agents, all ESBL-positive isolates carried the *tet*(A) gene, and three of them co-harbored the *tet*(B) gene. Sulfonamide resistance, detected in 12 isolates, was encoded by *sul1* (5 isolates), *sul2* (6 isolates), and *sul*3 genes (1 isolate). The two chloramphenicol-resistant isolates contained the *catA* gene, and the gentamicin-resistant isolate carried the *aac(3)-II* gene. Class 1 integrons were detected in 8 of the 12 trimethoprim/sulfamethoxazole-resistant ESBL producers (Table 4).

### 2.3. Molecular Typing and Conjugation Transference

Out of the 17 ESBL-producing isolates, 10 (58.8%) were found to belong to the D phylogenetic group. Six isolates (35.3%) were assigned to the B1 phylogroup, while only one isolate belonged to the B2 phylogroup (5.9%). MLST revealed seven different STs: ST23, ST38, ST48, ST117, ST131, ST1146, and ST5087 (Table 4).

Fifteen isolates (all bla_CTX-M-1_-harboring isolates and the bla_SHV-12_-harboring isolate) were found to be involved in the transferability of the plasmid-borne *bla*_CTX-M_ and *bla*_SHV-12_, respectively. Transconjugants that carried the *bla*_CTX-M-1_ gene acquired the IncK plasmid in five isolates and the IncI1 plasmid in association with tetracycline resistance encoded by *tet*(A) in one isolate. However, the conjugative plasmids in the remaining transconjugants were non-typable (Table 5).

## 3. Discussion

Avian colibacillosis is a bacterial infection affecting poultry farming, and antimicrobial-resistant APEC strains have been continuously reported globally. In Algeria, several studies have reported the occurrence of ACL in broilers nationwide [21,22,23,24]. Other authors have investigated the virulence determinants of APEC strains [18,19]. However, data on the AR genetic traits of APEC strains, notably ESBL-APEC isolates, are still lacking, and the available figures have been sourced from a sparse number of surveys [20]. To fill this gap, this study was conducted to provide a better molecular characterization of ESBL-producing APEC isolates collected in northeast Algeria throughout a seven-year period (2016–2021).

Out of the 248 liver samples collected from broilers and turkeys with ACL lesions at four slaughterhouses, 211 (85.1%) contained APEC isolates (isolation rates of 86% in broilers and 78.8% in turkeys). These findings corroborate previous studies conducted nationally [21,22,23,24] and internationally [25], in which the APEC isolation rates exceeded 80%. Our study provides more evidence that ACL remains one of the most prevailing avian diseases encountered in broilers and turkeys and a primary cause of the seizure and destruction of carcasses in slaughterhouses, as well as that *E. coli* is the major etiological agent in Algeria and across the world [26]. It is important to mention that the small sample size of turkeys is attributed to the limited number of turkey farms in the study region, which may generate several limitations.

In our study, 77.8% of APEC isolates belonged to three serogroups: O1 (31.3%), O2 (33.2%), and O78 (12.3%). These serogroups are the most often recovered amongst APEC isolates from cases of colibacillosis, with O78 being the most pathogenic [27,28,29]. In fact, the prediction of the pathogenicity of microorganisms isolated from sick animals is crucial. For *E. coli*, serotyping remains the basis of any pathogenicity diagnosis, even if it allows for the identification of a limited number of APEC isolates [30,31].

Our results are in line with those reported by numerous previous studies, but with variable frequencies. This variability could be attributed, on the one hand, to the use of different serotyping methods and, on the other hand, to the variable number of isolates studied from one study to another [9,27]. However, the distribution and frequencies of these dominant serogroups vary considerably geographically and temporally [32].

Also, it was reported that *E. coli* strains most commonly belonging to those serogroups possess typical APEC virulence factors such as lipopolysaccharide (LPS) (colibacillary endotoxin), the hemagglutinin protein Tsh, resistance to serum, and phagocytosis and have the ability to colonize the internal organs of chickens [27,33]. Likewise, possession of certain chromosomal or plasmid genes encoding virulence factors contributes significantly to APEC isolates’ biology and gives them a specific pathogenicity. Therefore, APEC strains may be a source of genes and plasmids that encode virulence factors [9,34].

Overall, 17 APEC isolates were resistant to CTX and/or CAZ. Basically, resistance to third-generation cephalosporins is a feature of *Enterobacteriaceae* that produce either ESBLs or AmpC β-lactamases, notably the CMY type. The latter are less prevalent than ESBLs but are still of great interest. In this study, all third-generation cephalosporin-resistant isolates were ESBL producers. Therefore, we only focused on ESBL genes, and no typical *Amp*C phenotype was detected.

The prevalence of ESBL-producing APEC in this study (8% from both avian species) seems much higher than the ones revealed in other studies, with rates of 5% and 1.9%, respectively [20,23]. Other previous surveys of poultry in Algeria investigating ESBL-producing *E. coli* carriage in fecal samples of healthy animals and broiler livers commercialized in the center of Algeria reported rates of 26.2% and 5.9%, respectively [13,35]. In Tunisia, a carriage rate of 25.5% of ESBL-producing *E. coli* was recorded in healthy chickens at laying hen farms [36]. Likewise, Misumi et al. [37] reported a percentage of 26.3% in chickens with ACL in Japan. Similar frequencies were observed in APEC causing ACL in broilers in Spain [38]. In Brazil, among 15 APEC isolates recovered from spiced chicken meat, 13 were ESBL producers [39]. Aside from the number of samples, the discrepancies in ESBL prevalence recorded in the different studies cited above could be traced back to the variability of the isolation protocols (with or without selective media supplemented with antibiotics).

The dramatically increasing rates of ESBL in avian *E. coli* strains could be primarily associated with the misuse of third-generation cephalosporins, among other β-lactams such as amoxicillin and ampicillin in poultry farms to control the early mortality caused by *E. coli* infections [40]. Ceftiofur, a third-generation cephalosporin that is indicated to treat cattle and swine respiratory infections, is not approved for use in poultry in many countries, including Algeria and the European Union. Nonetheless, numerous studies have propounded a possible link between the administration of ceftiofur and the expansion of third-generation cephalosporin-resistant and/or ESBL-producing *E. coli* strains in poultry [41,42,43]. Moreover, several surveys found that in certain poultry farms and hatcheries, Marek’s disease and avian influenza vaccines are administered jointly with ceftiofur to promote growth and prevent secondary bacterial infections [41,44].

Unsurprisingly, the *bla*_CTX-M_ group 1 subtypes—*bla*_CTX-M-1_ (n = 14) and *bla*_CTX-M-15_ (n = 2)—were the most predominant ESBL-encoding genes detected in our study. Indeed, it is well known that most outbreaks implicating ESBLs have been spawned by CTX-M subtypes rather than TEM or SHV. The two variants detected in this study are the most widely spread ESBL genes among animals and humans, respectively [15,17], and the CTX-M-1 enzyme is known to be the most frequent ESBL subtype in avian *E. coli* from healthy and sick chickens in Algeria and universally [20,45,46,47]. Throughout the globe, *bla*_CTX-M_-carrying isolates have been found in many ecological niches, including hospital and community settings, food-producing animals, wild animals, pets, food, and the environment [17,48]. The drastic explosion of CTX-M-type β-lactamases, also known as “the CTX-M pandemic”, might be the consequence of the dissemination of *bla*_CTX-M_ genes through mobile genetic platforms such as self-transmissible plasmids and transposons [49]. The *bla*_SHV-12_ gene was also retrieved in one specimen. SHV-producing *Enterobacteriaceae* have been previously reported in poultry and poultry products in different countries, including Algeria [13,50,51]. This low frequency of the SHV type among our APEC isolates confirmed that, together with the TEM ESBL types, SHV types are thought to have almost been displaced by CTX-M subtypes [49,52].

In this study, ESBL genes were carried by plasmids belonging to incompatibility groups IncK and IncI1. These two plasmids, referred to as epidemic plasmids, were successfully transferred through conjugation experiments, which demonstrate the significant role of horizontal gene transfer in the spread of ESBL genes and the genomic plasticity of our ESBL-producing APEC isolates.

Our ESBL-containing APEC isolates were distributed in three phylogroups: D (10 isolates), B1 (6 isolates), and B2 (1 isolate). These phylogroups differ considerably in terms of AR phenotype, genetic content, ecological niches, and pathogenicity [53]. While the B1 phylogroup is common in animals and encompasses commensal and intestinal pathogenic *E.coli* strains (InPEC), the D and B2 phylogroups comprise ExPEC strains, including greatly virulent ones [54,55]. Among the B1 phylogenetic group, four different STs—ST23, ST48, ST5087, and ST1146—were detected, underlining the diversity of our isolates. Previous reports indicated the circulation of the first three clones among avian *E. coli* isolates, highlighting the dissemination of these international lineages and their contribution to the wide spread of ESBL-encoding genes [13,56,57]. As for ST1146, it has been described to occur among *E. coli* isolates from poultry [58] and in grain culture soil samples [59]. Two genetic lineages assigned to the D phylogroup (ST38 and ST117) and one in the B2 phylogroup (ST131) were also observed in our isolates. ESBL-producing *E. coli* isolates of these lineages have been widely described across different ecologies and hosts [60,61], designating their role as ESBL drivers and high-risk clonal lineages.

Importantly, several studies have revealed the relationship between serotyping and STs in APEC isolates, with special attention paid to APEC O78 serotypes [12,62]. In our study, two ESBL-producing APEC isolates assigned to the O78 serogroup were sequenced as ST23. This finding is in line with previous reports that indicated that this serogroup is mainly represented by two distinct sequence types, ST23 and ST88 [62]. Also, the O78 ST117 APEC sequence type has largely spread and was implicated in large colibacillosis outbreak cases in Nordic broilers [63]. This lineage is known to be associated with high virulence and MDR determinants [64]. However, in our study, ESBL isolates with ST117 belonged to serotype O1. This could be explained by the fact that APEC O78 ST23 strains encode the H4 flagella antigens, while ST117 strains can encode a variety of O-antigens in place of O78, as mentioned by [64,65]. Genomic epidemiological studies, typing of H-antigens, and determination of virulence genes methods need to be further designed to comprehend the phylogenetic relationships of APEC types.

## 4. Materials and Methods

### 4.1. Research Approval

As this study was performed on dead animals, it was deemed to be exempt from needing protocol approval.

### 4.2. Sample Collection

**Figure 1:**
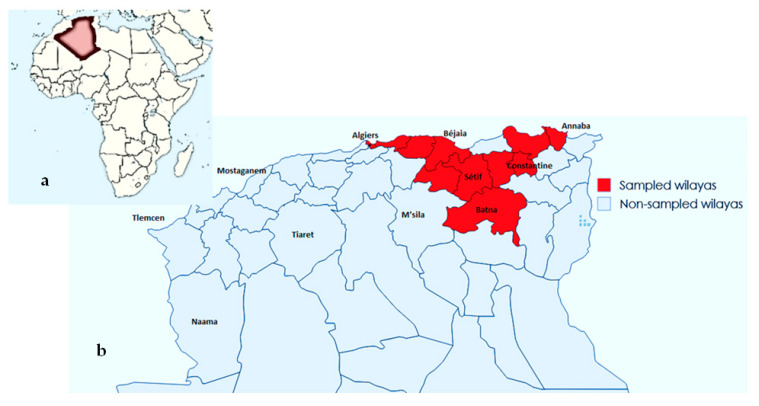
Distribution of poultry houses at cities level (Wilayas). (**a**): Map of Africa; (**b**): Locations of sampled poultry houses. Maps were established using https://www.mapsland.com and https://www.mapchart.net/, accessed on 12 September 2024.

From July 2016 to May 2021, 248 liver samples of condemned carcasses of 215 broilers and 33 turkeys diagnosed with ACL on *post mortem* examination carried out according to [66] were obtained at four licensed poultry slaughterhouses and one veterinary clinic in northeast Algeria, located in Setif, Algiers, Boumerdes, and Bordj Bou Arréridj (BBA) cities. As illustrated in Figure 1, live animals originated from 55 supply poultry houses located in 25 communes belonging to 11 wilayas in northeast Algeria (Sétif, Algiers, Batna, Béjaia, Tizi Ouzou, Boumerdes, Annaba, Constantine, Mila, Skikda, and Bordj Bou Arréridj). Liver samples were accurately labeled and then transported to the laboratory in cooled sterile containers to be processed.

### 4.3. Sample Processing and APEC Strain Isolation

A Bunsen burner was used to sterilize the surface of each organ sample before cutting it down sterilely into small cubes using sterile forceps and scissors. Enrichment was performed by placing some pieces of each organ sample in a 10 mL nutrient broth tube (Pasteur Institute of Algeria, PIA) and incubating overnight at 37 °C. Next, a drop of each enriched culture was streaked onto MacConkey selective and differential media (BiotechLab, Algiers, Algeria) and incubated at 37 °C for 24 h. Growth of *E. coli* gave rise to pink colonies (lactose-positive) often surrounded by precipitated bile salts. For each of the positive samples, one pure presumptive *E. coli* colony was picked up and identified using Gram staining, an oxidase test, a catalase test, and a triple sugar iron agar (TSI) test (PIA). Ultimately, API 20E system kits (BioMérieux, Marcy-l’Étoile, France) and the MALDI-TOF Biotyper system were used to identify *E. coli* isolates.

### 4.4. Serogrouping

According to the manufacturer’s instructions, APEC isolates were first streaked onto trypto-casein soy agar (TSA) media (Biolab, Algeria) and incubated at 37 °C for 24 h. Serogrouping was then carried out using a rapid agglutination slide (Citoglas, London, UK) and the antisera of the 3 O somatic groups (O1, O2, O78) (Biovac, Anger, France) as described by [67].

### 4.5. Antimicrobial Susceptibility and ESBL Testing

According to the guidelines of the Clinical Laboratory Standards Institute [68], the antimicrobial susceptibility of APEC isolates was determined using the Kirby–Bauer disk diffusion method. The following antimicrobial agents were used (μg/disk): ampicillin (AMP, 10), amoxicillin/clavulanic acid (AMC, 20 + 10), cefotaxime (CTX, 30), ceftazidime (CAZ, 30), imipenem (IMP, 10), nalidixic acid (NA, 30), ciprofloxacin (CIP, 5), neomycin (NEO, 30), gentamicin (GEN, 10), trimethoprim/sulfamethoxazole (SXT, 1.25 + 23.75), chloramphenicol (CLR, 30), tetracycline (TET, 30), and nitrofurantoin (FUR, 300). The quality control strain *E. coli* ATCC 25,922 was used for antimicrobial susceptibility testing.

Phenotypic ESBL screening was conducted for all APEC isolates through the double-disk synergy test (DDST), which uses cefotaxime and ceftazidime disks (third-generation cephalosporins) along with an amoxicillin/clavulanic acid disk (β-lactamase inhibitor) placed 30 mm apart, center to center [69]. ESBL-producing *K. pneumoniae* ATCC 700,603 were adopted as a reference strain. Additionally, the colistin minimum inhibitory concentration (MIC) which delineates in vitro levels of susceptibility or resistance was evaluated in ESBL-producing APEC isolates using the agar dilution method, as prescribed by the European Committee on Antimicrobial Susceptibility Testing guidelines [70].

### 4.6. Antimicrobial Resistance Genes in ESBL-Producing APEC Isolates

In ESBL-producing APEC isolates, the rapid boiling method was used to extract genomic DNA [71]. Afterwards, PCR and consecutive sequencing assays were used to investigate the presence of ESBL-encoding genes (*bla*_CTX-M_ groups, *bla*_TEM_, and *bla*_SHV_), colistin resistance genes (*mcr-1* to *mcr-3*), tetracycline resistance genes (*tet*(A) and *tet*(B)), sulfonamides resistance genes (*sul1*, *sul2,* and *sul3*), gentamicin resistance genes (*aac(3)-I* and *aac(3)-II*), and chloramphenicol resistance genes (*cml*A, and *flor*R and *cat*A). Moreover, the detection of class 1 and 2 integrons was screened through the presence of integrase genes (*intI* and *intII*) [13,72]. The beta-lactamase genes obtained by PCR (*bla*_CTX-M_ groups, *bla*_TEM_, and *bla*_SHV_) were sequenced to determine the variants of the genes [13,72].

### 4.7. Phylogrouping, MLST, and Conjugation Experiments

ESBL-producing APEC isolates were categorized into one of the four phylogenetic groups (A, B1, B2, or D) using a rapid protocol previously described [8]. Multilocus sequence typing (MLST) was used to characterize selected ESBL-producing APEC isolates using the sequences of internal fragments of the seven house-keeping genes specific to *E. coli* (*adk*, *fum*C, *gyr*B, *icd*, *mdh*, *pur*A and *rec*A). The sequence type (ST) of each isolate was then determined using the *E. coli* MSLT database (https://enterobase.warwick.ac.uk/species/ecoli/allele_st_search, accessed on 20 April 2019).

Conjugation experiments were performed to determine the transferability of the previously retrieved ESBL-encoding genes. Selected ESBL-harboring APEC isolates were selected as donors, and *E. coli* CSH26 (plasmid-free, lactose-negative, and Rif^R^) was chosen as recipient. Rifampicin (50 µg/mL) and cefotaxime (4 µg/mL) were added to MacConkey agar to select transconjugants, which were subsequently subjected to antimicrobial susceptibility testing and ESBL gene amplification using PCR assays. Ultimately, PCR-based replicon typing (PBRT) of the donors and the ESBL-containing transconjugants was carried out to look into the acquired genes [13,73].

## 5. Conclusions

In this study, we report the high prevalence of O2 and O1 serogroups among APEC isolates from Algerian poultry and the dominance of the *bla*_CTX-M-1_-encoding gene in ESBL-producing APEC isolates. MLST showed a great diversity of ESBL-producing APEC isolates, with detection of high-risk clonal lineages. We also report the detection of successful transferred epidemic plasmids (IncK and IncI1), highlighting the genomic plasticity of our ESBL-producing APEC isolates. Concerted efforts from all poultry actors are needed to establish vigilant infection monitoring strategies and mitigate the spread of ESBL-producing APEC strains in Algeria.

## Figures and Tables

**Table 1:** Serogroups of broiler and turkey APEC isolates (n = 211).

Serogroup	Broiler Isolates (n = 185)		Turkey Isolates (n = 26)		Total Isolates (n = 211)	
O1	50 (27%)	143 (77.3%)	16 (61.5%)	21 (80.8%)	66 (31.3%)	164 (77.8%)
O2	66 (35.7%)		4 (15.4%)		70 (33.2%)	
O78	27 (14.6%)		1 (3.8%)		28 (13.3%)	
Non-Typable isolates	42 (22.7%)		5 (19.2%)		47 (22.2%)	

**Table 2:** Antimicrobial resistance rates of broiler and turkey APEC isolates (n = 211).

Antibiotic Disks Used	Broiler APEC Isolates (n = 185)	Turkey APEC Isolates (n = 26)	Total APEC Isolates n = 211
Ampicillin	150 (81.1%)	26 (100%)	176 (83.4%)
Amoxicillin/clavulanic acid	140 (75.7%)	18 (69.2%)	158 (74.9%)
Cefotaxime	12 (6.5%)	5 (19.2%)	17 (8%)
Ceftazidime	2 (1.1%)	1 (3.8%)	3 (1.4%)
Imipenem	0 (0%)	0 (0%)	0 (0%)
Nalidixic acid	175 (94.6%)	25 (96.1%)	200 (94.8%)
Ciprofloxacin	121 (65.4%)	19 (73%)	140 (66.3%)
Neomycin	63 (34.1%)	8 (30.8%)	71 (33.6%)
Gentamicin	11 (5.9%)	3 (11.5%)	14 (6.6%)
Trimethoprim/sulfamethoxazole	140 (75.7%)	23 (88.5%)	163 (77.2%)
Chloramphenicol	36 (19.5%)	11 (42.3%)	47 (22.3%)
Tetracycline	181 (97.8%)	26 (100%)	207 (98.1%)
Nitrofurantoin	65 (35.1%)	10 (38.5%)	75 (35.5%)

**Table 3:** Multidrug resistance in broiler and turkey APEC isolates (n = 211).

Antimicrobial Classes with Resistance	Broiler APEC Isolates (n = 185)		Turkey APEC Isolates (n = 26)	
	Number of Isolates	Prevalence (%)	Number of Isolates	Prevalence (%)
0	2	1.1	0	0
1	0	0	0	0
2	9	4.9	0	0
3 *	37	20	3	11.5
4 *	51	27.6	8	30.8
5 *	42	22.7	9	34.6
6 *	30	16.2	5	19.2
7 *	14	7.6	1	3.8
Total	185	100	26	100%

*: MDR (multidrug resistance).

**Table 4:** Genetic characterization of ESBL-producing APEC isolates (n = 17).

ESBL Isolate Code	Poultry House Location	Sampling Point (Year)	Breed Type (Age)	Serogroup	Phylogroup/ST	Antimicrobial Resistance Phenotype	MIC of COL (µg/µL)	Antimicrobial Resistance Genotype
C8195	Djemila (Setif)	Slaughterhouse (2016)	Broiler (7–8 weeks)	O2	B1/ST5087	AMP-AMC-CTX-SXT-FUR-CLR-NAL-CIP-TET	≤1	*bla*_CTX-M-1_, *tet(A)*, *Intl1*, *Cat*, *sul2*
C8196	Hamma Bouziane (Constantine)	Slaughterhouse (2016)	Broiler (7–8 weeks)	O2	B1/ST48	AMP-CTX-SXT-TET	≤1	*bla*_CTX-M-1_, *tet(A)*, *Intl1*, *sul2*
X800	Ain Arnat (Setif)	Slaughterhouse (2016)	Broiler (7–8 weeks)	O1	D/ST38	AMP-AMC-CTX-SXT-NAL-CIP-TET	≤1	*bla*_CTX-M-1_, *tet(A*), *Intl1*, *sul2*
X801 X802 X803	Bejaia	Slaughterhouse (2016)	Broilers (7–8 weeks)	O78 O78 O1	B1/ST23 B1/ST23 B1/ST23	AMP-AMC-CTX-SXT-NAL-CIP-TET AMP-AMC-CTX-NAL-TET AMP-AMC-CTX-NAL-TET	≤1	*bla*_CTX-M-1_, *tet(A)*, *sul1* *bla*_CTX-M-1_, *tet(A)* *bla*_CTX-M-1_, *tet(A)*
X804	Remada (Setif)	Veterinary clinic (2018)	Turkey (12 weeks)	O2	B2/ST131	AMP-CTX-GEN-SXT-CLR-NAL-CIP-TET	≤1	*bla*_CTX-M-1_, *tet(A)*, *Intl1*, *cat*, *sul1, aac(3)-II*
X805 X806	Bir Hadada (Setif)	Veterinary clinic (2018)	Broilers (3 weeks)	O1 O1	D/ST117 D/ND	AMP-CTX-SXT-NAL-CIP-TET AMP-CTX-SXT-NAL-CIP-TET	≤1	*bla*_CTX-M-1_, *bla*_TEM-1_, *tet(A)*, *tet(B)*, *Intl1*, *sul1* *bla*_CTX-M-1_, *bla*_TEM-1_, *tet(A)*, *tet(B)*, *Intl1*, *sul2*
X807 X808	Bellaa (Setif)	Veterinary clinic (2018)	Turkeys (4 weeks)	O1 O1	D/ST117 D/ST117	AMP-CTX-SXT-NAL-TET AMP-CTX-SXT-NAL-CIP-TET	≤1	*bla*_CTX-M-1_, *bla*_TEM-1_, *tet(A)*, *tet(B)*, *Intl1*, *sul2* *bla*_CTX-M-1_, *bla*_TEM-1_, *tet(A)*, *Intl1*, *sul2*
X809 X810	Bir Hadada (Setif)	Veterinary clinic (2018)	Broilers (5 weeks)	O1 O1	D/ST117 D/ND	AMP-CTX-NAL-TET AMP-CTX-NAL-TET	≤1	*bla*_CTX-M-1_, *tet(A)*, *bla*_CTX-M-1_, *tet(A)*
X811	Bir Hadada (Setif)	Veterinary clinic (2018)	Turkey (7 weeks)	O1	D/ST117	AMP-CTX-NAL-TET	≤1	*bla*_CTX-M-1_, *tet(A)*
X1241	Beida Bordj (Setif)	Slaughterhouse (2018)	Turkey (7–8 weeks)	NT	B1/ST1146	AMP-CTX-CAZ-NEO-SXT-TET	≤1	*bla*_SHV-12_, *bla*_TEM-1_, *tet(A)*, *sul3*
BBA001	(Bordj Bou Arréridj)	Slaughterhouse (2021)	Broiler (7–8 weeks)	NT	D/ND	AMP-CTX-CAZ-TET-SXT	≤1	*bla*_CTX-M-15_, *tet(A*), *sul1*
BBA002	(Bordj Bou Arréridj)	Slaughterhouse (2021)	Broiler (7–8 weeks)	NT	D/ND	AMP-CTX-CAZ-TET-SXT	≤1	*bla*_CTX-M-15_, *tet(A)*, *sul1*

AMP: ampicillin; AMC: amoxicillin/clavulanic acid; CTX: cefotaxime; CAZ: ceftazidime; COL: colistin; NAL: nalidixic acid; CIP: ciprofloxacin; GEN: gentamicin; NEO: neomycin; SXT: trimethoprim/sulfamethoxazole; FUR: nitrofurantoin; CLR: chloramphenicol; TET: tetracycline; MIC: minimum inhibitory concentration; ESBL: extended-spectrum β-lactamase; ST: sequence type; NT: non-typable; ND: not determined.

**Table 5:** Conjugation in ESBL-producing APEC isolates (n = 17).

ESBL Isolate (Donor)	Conjugation/Plasmids in Donors	Transconjugants	Antimicrobial Resistance Phenotype in TC	Plasmids/Antimicrobial Resistance Genotype in TC
C8195	+/IncI1, FIB, FIC, IncK	X1223	ESBL, RIF ^R^	IncK/*bla*_CTX-M-1_
C8196	+/IncI1, IncK	X1224	ESBL, RIF ^R^	IncK/*bla*_CTX-M-1_
X800	+/IncI1	X1225	ESBL, RIF ^R^, TET ^R^	IncI1/*bla*_CTX-M-1_, *tet(A)*
X801	+/IncI1, FIB, IncK	X1468	ESBL, RIF ^R^	IncK/*bla*_CTX-M-1_
X802	+/IncI1, FIB, IncK	X1226	ESBL, RIF ^R^	IncK/*bla*_CTX-M-1_
X803	+/IncI1, FIB	X1469	ESBL, RIF ^R^	IncK/*bla*_CTX-M-1_
X804	+/IncI1, FIB	X1227	ESBL, RIF ^R^, GEN ^R^	Non-typable/*bla*_CTX-M-1_, *aac(3)-II*
X805	+/IncI1, FIB, FIC	X1228	ESBL, RIF ^R^	Non-typable/*bla*_CTX-M-1_
X806	+/IncI1, FIB	X1470	ESBL, RIF ^R^	Non-typable/*bla*_CTX-M-1_
X807	+/IncI1, FIB, FIC	X1229	ESBL, RIF ^R^	Non-typable/*bla*_CTX-M-1_
X808	+/IncI1, FIB	X1471	ESBL, RIF ^R^	Non-typable/*bla*_CTX-M-1_
X809	+/IncI1, FIB	X1472	ESBL, RIF ^R^	Non-typable/*bla*_CTX-M-1_
X810	+/IncI1, FIB	X1473	ESBL, RIF ^R^	Non-typable/*bla*_CTX-M-1_
X811	+/IncI1, FIB	X1474	ESBL, RIF ^R^	Non-typable/*bla*_CTX-M-1_
X1241	+/K, IncI1	X1475	ESBL, RIF ^R^	Non-typable/*bla*_SHV-12_
BBA001	−	**/**	/	/
BBA002	−	**/**	/	/

+: positive conjugation; −: negative conjugation; TC: transconjugants; RIF: rifampicin; TET: tetracycline; GEN: gentamicin; R: resistance.

## Data Availability

The original contributions presented in this study are included in the article; further inquiries can be directed to the corresponding author.
